# Vitamin D3 and Body Composition Association with Graft Function in Long-Term Kidney Transplant Recipients

**DOI:** 10.3390/ijms27125384

**Published:** 2026-06-15

**Authors:** Maksymilian Hryciuk, Zbigniew Heleniak, Sylwia Małgorzewicz, Fabian Halleck, Alicja Dębska-Ślizień, Klemens Budde

**Affiliations:** 1 Department of Nephrology, Medical University of Gdańsk, Dębinki 7 Street, 80-211 Gdańsk, Poland; sylwia.malgorzewicz@gumed.edu.pl (S.M.); alicja.debska-slizien@gumed.edu.pl (A.D.-Ś.); 2 Department of Clinical Nutrition, Medical University of Gdańsk, Dębinki 7 Street, 80-211 Gdańsk, Poland; 3 Medizinische Klinik mit Schwerpunkt Nephrologie und Internistische Intensivmedizin Charité, Universitätsmedizin Berlin, 10117 Berlin, Germany; fabian.halleck@charite.de (F.H.); klemens.budde@charite.de (K.B.)

**Keywords:** kidney transplant, 25(OH)D_3_, 1,25(OH)_2_D_3_, eGFR, graft function, body composition, BMI

## Abstract

This study evaluated the association between vitamin D3 levels, transplanted kidney function, and body composition in 315 stable renal transplant recipients (median 7.7 years post-transplant). The biochemical profile included eGFR, PTH, calcium, phosphorus, and 25(OH)D_3_ levels. Vitamin D status was defined as deficiency (<20 ng/mL), insufficiency (20–30 ng/mL), or optimal (>30 ng/mL). Body composition was assessed via bioelectrical impedance analysis, capturing parameters such as BMI, visceral fat area, and phase angle. Multivariable quantile regression models were used to assess the associations between clinical/metabolic parameters and graft function. Vitamin D3 supplementation was prescribed in 61.5% of patients, with 49.7% receiving active analogues and 50.3% cholecalciferol. Results showed that 25(OH)D_3_ levels did not correlate with graft function in the total population, and no significant differences in eGFR were observed regarding vitamin D status. In multivariable models, 25(OH)D_3_ levels correlated significantly only with calcium levels. No significant correlations were observed between vitamin D and transplant vintage, age, eGFR, or any anthropometric and body composition parameters.

## 1. Introduction

Kidney transplantation remains the gold standard and the most effective treatment for end-stage kidney disease, offering patients significantly better quality and length of life, as well as lower costs compared to dialysis therapy [1,2]. In Europe, according to the European Renal Association Registry data from 2023, the prevalence of renal replacement therapy (RRT) was 1188 patients per million population, corresponding to approximately 630,000 individuals receiving RRT. Among these patients, 47% (around 296,000 individuals) were living with a functioning kidney transplant; this means that nearly half of all Europeans requiring RRT benefit from kidney transplantation [3]. Globally, it is estimated that over 2 million patients are living with a functioning kidney transplant [4].

However, despite surgical success and advances in immunosuppressive therapy, graft function in kidney transplant recipients (KTRs) may deteriorate over time, highlighting the need for meticulous multidisciplinary post-transplant care. This decline may result from both immunological and non-immunological factors, including drug nephrotoxicity, infections, recurrent primary disease, ischemic injury, and metabolic or cardiovascular complications [5]. One of the potential, yet often underestimated, factors associated with post-transplant metabolic outcomes is vitamin D deficiency, which is commonly observed in KTRs [6,7]. The causes of vitamin D deficiency after kidney transplantation include pre-existing chronic kidney disease–mineral bone disorders (CKD-MBD), limited exposure to ultraviolet radiation due to skin cancer prevention strategies, and the effects of immunosuppressive regimens (e.g., steroids and calcineurin inhibitors) which enhance vitamin D catabolism or suppress receptor expression [8]. The primary source of vitamin D is cutaneous synthesis under ultraviolet B exposure. Cholecalciferol is then converted in the liver to 25(OH)D and subsequently in the kidneys to its active form, 1,25(OH)_2_D_3_ (calcitriol). While successful kidney transplantation partially restores the renal 1-alpha-hydroxylase activity required for this conversion, the process often remains suboptimal compared to in healthy individuals. Vitamin D3 is responsible for intestinal absorption of phosphate and calcium, and bone mineralization and also plays a role in modulating the immune system [9].

Vitamin D may support graft function through immunomodulatory effects, including suppression of proinflammatory immune responses and promotion of immune tolerance [10,11]. It may also modulate renin–angiotensin–aldosterone system activity, thereby influencing blood pressure and renal perfusion [12]. Moreover, there is evidence that vitamin D may protect endothelial cells and reduce fibrosis in the renal cortex, which is crucial for preserving the function of nephrons [13].

Vitamin D, as a regulator of calcium–phosphate metabolism, may influence calcium deposition in soft tissues, which is relevant to vascular calcification and microcirculation in the transplanted kidney [14]. Low vitamin D levels have also been associated with higher BMI and insulin resistance, both of which may adversely affect graft function [15]. Numerous observational studies have shown that RTRs with vitamin D deficiency tend to have poorer graft survival [16,17,18]. However, clinical trial results remain inconclusive, highlighting the need for further research.

The unique objective of this study, conducted under real-world clinical conditions, was to simultaneously evaluate the relationship between both circulating 25(OH)D_3_ and active 1,25(OH)_2_D_3_ levels, and long-term kidney graft function. This study uniquely correlates vitamin D status with advanced body composition parameters, factoring in the time since transplantation. Such an analysis, reflecting pragmatic outpatient post-transplant care, aims to provide precise clinical context regarding the complex metabolic associations of vitamin D in long-term kidney transplant recipients.

## 2. Results

### 2.1. Study Population Characteristics

A total of 315 RTRs were included in the analysis and stratified into three groups based on serum 25(OH)D_3_ levels: deficiency (*n* = 100), suboptimal status (*n* = 103), and optimal status (*n* = 112). The mean age of the cohort was 52.3 ± 13.7 years, 61% were male, and the median time since transplantation was 6.0 years (IQR 2.3–11.0), with no significant differences in these demographic parameters across the three vitamin D categories. Clinical comorbidities—including diabetes, hypertension, cardiovascular disease, heart failure, and a history of malignancy—were highly prevalent but comparably distributed among the groups. Similarly, routine laboratory parameters (hemoglobin, C-reactive protein, and serum albumin) and the primary etiologies of end-stage renal disease (with glomerulonephritis being the most common) did not differ significantly between the cohorts. According to BMI criteria, overweight and obesity were identified in 35.6% and 14.9% of the entire cohort, respectively, presenting a homogenous distribution regardless of vitamin D status. Detailed baseline clinical, laboratory, and anthropometric characteristics are presented in Table 1 and Table 2.

### 2.2. Vitamin D, Calcium Supplementation, and Immunosuppressive Therapy

Tacrolimus was the most used calcineurin inhibitor across all vitamin D categories, followed by cyclosporine, with no significant differences between groups. Glucocorticoid use was also comparable. The distribution of immunosuppressive treatment across vitamin D status groups is shown in Table 3.

Vitamin D supplementation was prescribed in 193 patients (61.3%). Among them, 41.5% (*n* = 80) received active vitamin D analogues (calcitriol, alfacalcidol, or paricalcitol), 46.6% (*n* = 90) were treated with cholecalciferol, and 11.9% (*n* = 23) received both therapies. The prevalence of vitamin D supplementation significantly increased across the status categories, from 43.0% (*n* = 43) in the deficient group to 60.0% (*n* = 62) and 78.6% (*n* = 88) in the suboptimal and optimal groups, respectively (*p* < 0.0001). Concurrently, the use of calcium carbonate (mean dose 1700 mg/day) and cinacalcet differed significantly among the groups, with both therapies being heavily concentrated in the extreme (deficient or optimal) categories due to underlying mineral disorders.

### 2.3. Vitamin D Status and Graft Function, Biochemical Parameters, and Body Composition

Graft function did not differ significantly across vitamin D status groups (*p* = 0.16; Table 2) as well as calcium and phosphate levels. PTH levels, which differed significantly by vitamin D, were highest in the deficient group compared to those with suboptimal or optimal levels (*p* < 0.0001) (Table 2). Among the analyzed variables, a statistically significant positive correlation was found between 1,25(OH)_2_D_3_ and 25(OH)D_3_ levels (R = 0.133, *p* = 0.018), whereas PTH levels were inversely correlated with vitamin D levels (R = –0.298, *p* < 0.0001).

No significant differences were observed in anthropometric or body composition parameters, including BMI, WC, WHR, VFA, BFM, SLM, and PA, across vitamin D status groups (all *p* > 0.05; Table 2).

**Figure 1:**
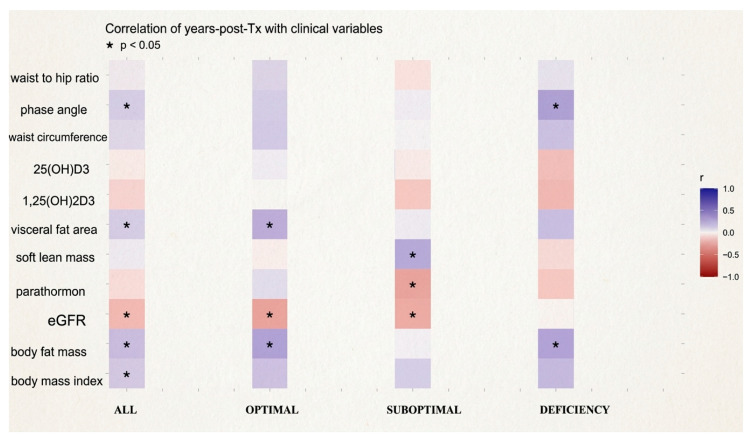
Correlation between time since kidney transplantation and clinical variations.

Time since transplantation did not correlate with 25(OH)D_3_ or 1,25(OH)_2_D_3_ levels, showing similar patterns across all vitamin D status groups. However, a longer time since transplantation was significantly associated with poorer graft function (lower eGFR) and higher adiposity parameters, including BMI, VFA, BFM, and PA (Figure 1).

### 2.4. Multivariable Regression

Multivariable quantile regression analysis showed that age, serum phosphorus, and PTH levels were consistently and negatively associated with eGFR across all quantiles. In contrast, serum 1,25(OH)_2_D_3_ was positively associated with eGFR, particularly in the lower and median quantiles. Serum 25(OH)D_3_ showed only a weak inverse association limited to the lowest quantile. Among body composition parameters, only SLM was negatively associated with eGFR in the upper quantile, while other anthropometric indices, immunosuppressive therapy, calcium level, and time since transplantation showed no significant associations (Table 4).

Multivariable linear regression analysis identified serum calcium as the only independent predictor of 25(OH)D_3_ levels. Higher calcium concentrations were associated with lower 25(OH)D_3_ levels (β = −30.553; 95% CI: −52.72 to −8.39; *p* = 0.007). No significant associations were found for age; time since transplantation; eGFR; PTH; phosphorus; immunosuppressive regimen; or body composition parameters, including BMI, WHR, VFA, and PA (Table 5).

Overall, these findings suggest that graft function in this cohort was mainly associated with age, mineral metabolism, and active vitamin D levels, whereas body composition parameters showed limited clinical relevance.

## 3. Discussion

According to our knowledge, this is the first large-scale study to evaluate vitamin D status (25 OH and 1,25 OH), detailed body composition, and their relationship with graft function in stable KTRs many years after transplantation, with patients stratified according to clearly defined vitamin D categories reflecting real-life patient care and therapeutic management. This analysis revealed that 25(OH)D_3_ levels did not correlate with graft function, time after kidney transplant, or body composition parameters, but higher 25(OH)D_3_ levels were associated with lower PTH concentrations. In the multivariable models, renal function was associated with age, phosphorus levels, and active vitamin D (1,25(OH)_2_D_3_) but not with 25(OH)D_3_ or body composition (BMI, WHR, WC, VFA, BFM, SLM, and PA). In contrast to the study by Grzejszczak et al. [19], who reported a significant negative correlation between fat tissue index and 25(OH)D levels, our study on a larger cohort (*n* = 315) did not observe such an association. Moreover, our study offers a more robust and comprehensive analysis of the interplay between vitamin D, PTH, body composition, and long-term graft function.

### 3.1. Vitamin D Status and Graft Function

In the present cohort, serum 25(OH)D_3_ concentrations did not differ across eGFR categories and were not independently associated with graft function in multivariable analyses. Similar findings have been reported in previous research, including the large randomized VITALE trial, where markedly higher vitamin D levels achieved after cholecalciferol supplementation were not associated with improved graft function (mean eGFR 51 vs. 54 mL/min/1.73 m^2^ in high- vs. low-dose groups). That study included patients at a median of 25 months (12–24 months) post-transplantation yet failed to account for detailed body composition metrics beyond BMI as potential confounding factors. In our stable post-transplant population, no significant differences were observed in kidney function across vitamin D3 status groups. Taken together, these data suggest that vitamin D status may not be directly associated with long-term kidney graft function [20]. Furthermore, similar findings have been reported in previous research. In the study by Tsujita et al., cholecalciferol supplementation was evaluated in KTRs, who used it from 1 month post-transplant and up to 12 months; in contrast to our data, it focused on the early post-transplant period. After supplementation, median eGFR values were similar between the cholecalciferol and placebo groups throughout the study, remaining around 46–48 mL/min/1.73 m^2^, indicating no significant effect of vitamin D3 supplementation on renal graft function during the first year post-transplant [21]. Although the above-cited trials did not demonstrate an association between vitamin D status and eGFR, our study—conducted at a mean of 7.66 years post-transplant—similarly did not provide new evidence regarding this issue. Interestingly, in our multivariable quantile regression analysis, 25(OH) D_3_ deficiency was paradoxically associated with higher eGFR in the median and upper quantiles, although not in the lower range. We consider this unexpected finding more likely related to residual confounding, reverse causality, or differences in clinical practice rather than a true protective effect of vitamin D deficiency. In routine post-transplant care, patients with worse or declining graft function are usually monitored more closely and are more likely to receive vitamin D supplementation. As a result, patients with better kidney function may remain untreated and therefore appear as “vitamin D-deficient” in a single cross-sectional assessment. Importantly, this pattern contrasts with most available evidence, where lower vitamin D concentrations have been linked to poorer renal outcomes [22]. In contrast to 25(OH)D_3_, 1,25(OH)_2_D_3_ serum levels were positively associated with eGFR, particularly in the lower and median quantiles.

This association reflects a unidirectional causal pathway, where functioning renal mass determines 1-alpha-hydroxylase activity and calcitriol production. Preserved graft function naturally ensures a higher capacity for endogenous hormone production. Given our cross-sectional design, these findings describe metabolic associations at a single time point and do not imply reverse causality (i.e., active vitamin D driving eGFR improvement).

Although our cross-sectional analysis did not demonstrate a significant association between graft function and 25(OH)D_3_, evidence from longitudinal and prospective studies points toward a different relationship, as shown in Japanese cohort of 264 RTRs, where low baseline 25-hydroxyvitamin D (25(OH)D) levels predicted a faster decline in eGFR, particularly within 10 years post-transplant and independently of other confounders. This association weakened in patients with longer transplant vintage, more than ten years [23]. Similarly, a cross-sectional study from a low-latitude city demonstrated that lower 25(OH)D status correlated with decreased eGFR and increased proteinuria, even after adjusting for body fat and conventional cardiovascular risk factors [24].

Taken together, these findings suggest that the relationship between vitamin D status and graft function may depend on transplant vintage and may be partially mediated by the immunoregulatory properties of vitamin D. Although our cross-sectional study failed to detect a straightforward association and even yielded a paradoxical signal in regression modeling, vitamin D3 insufficiency may nevertheless co-exist with progressive graft dysfunction over time, particularly in the early post-transplant years—a concern that is especially important given the very high prevalence of vitamin D deficiency in RTRs, affecting up to 90% of patients [7]. Moreover, the lack of association in a stable, long-term RTR cohort, as in our study, may reflect a plateau in graft function where transient metabolic effects of vitamin D are attenuated. This underscores that cross-sectional designs may underestimate temporal and mechanistic links that only become apparent in prospective or interventional frameworks.

### 3.2. Vitamin D and Mineral Metabolism

In our study population, vitamin D3 deficiency was strongly associated with significantly elevated serum PTH levels, whereas patients with optimal vitamin D3 status demonstrated markedly lower PTH concentrations. This inverse relationship is consistent with the well-established role of vitamin D in calcium–phosphate homeostasis and secondary hyperparathyroidism after kidney transplantation [25,26]. Similar findings were reported in large observational studies, where vitamin D insufficiency correlated with higher PTH levels and disturbances in mineral metabolism among RTRs [27]. Furthermore, our data showed a trend toward higher serum 1,25(OH)_2_D_3_ concentrations in the suboptimal and optimal groups compared with the deficiency group, although this difference did not reach statistical significance. This observation is biologically justified, as sufficient substrate (25(OH)D) availability is required for renal 1α-hydroxylase activity and subsequent production of 1,25(OH)_2_D_3_ [28,29]. Previous studies have similarly suggested that vitamin D repletion may enhance circulating active vitamin D metabolites in transplant recipients [16]. A positive correlation between 25(OH)D_3_ and 1,25(OH)_2_D_3_ indicates that cholecalciferol (D_3_) supplementation is effective in improving the active metabolite despite immunosuppressive therapy [30], an argument for using classical vitamin D3 rather than only active forms, which is reflected in the new European Renal Association (ERA) recommendations [31]. In multivariable quantile regression analyses, elevated PTH and phosphorus were consistently associated with lower eGFR, whereas higher 1,25(OH)_2_D_3_ levels were associated with better graft function, emphasizing the importance of the vitamin D–PTH axis rather than circulating 25(OH)D_3_ alone [32,33]. In summary, our findings indicate that 25(OH)D_3_ indirectly regulates mineral metabolism by suppressing PTH and providing a substrate for the synthesis of 1,25(OH)_2_D_3_, which in turn appears to be positively associated with graft function. This highlights the complex interplay between vitamin D status, PTH, and active vitamin D in long-term kidney transplant outcomes [27]. We stratified our cohort based on 25(OH)D_3_ rather than 1,25(OH)_2_D_3_ because major clinical guidelines (KDIGO and ERA) establish 25(OH)D_3_ as the gold standard for defining vitamin D status [26,31]. Moreover, measuring 1,25(OH)_2_D_3_ is highly complex compared to 25(OH)D_3_ as it circulates in the picomolar range—at concentrations approximately 1000 times lower than its precursor—and currently lacks a reference method [31]. Furthermore, in line with our pragmatic, ‘real-world’ study design, the cohort inherently includes patients on heterogeneous maintenance regimens (cholecalciferol, alfacalcidol, and paricalcitol). While this introduces pharmacological diversity, maintaining an unselected cohort accurately reflects routine outpatient post-transplant care.

### 3.3. Body Composition, Transplantation Vintage, and Vitamin D Status

Our cohort analysis demonstrated that over time, following kidney transplantation, patients gradually increased their body weight, BMI, BFM, visceral fat area, and PA. These observations suggest that long-term KTRs are at risk of developing obesity—a simultaneous increase in fat tissue with loss of muscle mass and function—as well as progressive changes in body composition. Similar findings have been reported in other studies, where the increase was primarily driven by gains in fat mass [34,35]. This phenomenon may result from several factors, including reduced physical activity due to chronic treatment and post-transplant caution, metabolic alterations associated with long-term immunosuppressive therapy (particularly glucocorticoids and certain calcineurin inhibitors), chronic low-grade inflammation, and hormonal changes related to age and chronic kidney disease [36,37,38]. Despite these anthropometric changes, no significant associations were observed between time since transplantation and serum 25(OH)D_3_ or 1,25(OH)_2_D_3_ levels, in contrast to the results of Bienaimé et al. [16], who reported that patients in the long-term post-transplant period had slightly higher 25(OH)D_3_ levels, although other studies, such as Priyadarshini et al. [39], observed changes primarily in 1,25(OH)_2_D_3_ rather than 25(OH)D_3_ over time. Our findings suggest that vitamin D deficiency in RTRs does not result directly from the passage of time or changes in body composition but rather from other factors such as limited sunlight exposure, age, diet, or the type of immunosuppressive regimen [27,40]. No significant associations were observed between classical anthropometric parameters (BMI, WHR, WC, BFM, SLM, and PA) and graft function measured by eGFR in our study. Similarly, anthropometric measures did not significantly correlate with serum 25(OH)D_3_ or 1,25(OH)_2_D_3_ concentrations. These results suggest that vitamin D deficiency in RTRs and renal graft function are not directly related to body composition. Nevertheless, Grzejszczak et al., in stable RTRs (~5–6 years post-transplant), also did not find an association between BMI or body weight and 25(OH)D_3_, although they did demonstrate an inverse correlation between fat tissue index and 25(OH)D_3_, and a positive one with lean mass [19]. In contrast, Baxmann et al. reported a significant association between BMI and 25(OH)D_3_, indicating that excessive body weight and increased fat content contribute to hypovitaminosis D in RTRs [41]. Similarly, Argentino et al. did not confirm a general correlation between anthropometry and 25(OH)D but observed that in women, the 25(OH)D/body weight ratio was inversely related to BMI, WC, and fat percentage [42].

In summary, our findings confirm that there is no clear or consistent relationship between classical anthropometric parameters and serum vitamin D3 or eGFR. Literature data suggest that it is rather specific aspects of body composition—particularly high fat mass and low lean mass—that may modulate vitamin D status. This may explain why correlations are observed in some studies or specific patient subgroups but disappear when analyzing entire cohorts. In our cohort, we did not observe significant associations between classical anthropometric parameters and kidney function as measured by eGFR. However, the literature on this subject is heterogeneous: some studies indicate that detailed measures of excess adiposity, such as percent body fat or computed tomography (CT)-measured visceral fat areas, are associated with poorer kidney function, whereas analyses based solely on BMI often yield less definitive results [43,44].

Moreover, data from the literature suggest that changes in fat and fluid compartments may coincide with a decline in eGFR, and a higher proportion of body fat measured by quantitative methods correlates with lower GFR in other cohorts [43,45,46]. On the other hand, meta-analyses and analytical studies indicate heterogeneity of evidence regarding the impact of BMI on renal excretory function and clinical outcomes [44,47]. The absence of a significant and reproducible correlation between classical anthropometric parameters and eGFR in our study is consistent with the wide range of results reported in previous publications. However, it should be noted that studies in this area among RTRs are very limited. This scarcity of data highlights the need for more precise body composition assessment methods (e.g., dual-energy X-ray Absorptiometry, and CT-based measurements of visceral fat) and long-term analyses that could better capture the potential impact of body composition on graft function. Consequently, conclusions often require supplementation with or extrapolation from studies conducted in other cohorts, such as patients with chronic kidney disease. In addition, we observed that serum 1,25(OH)_2_D_3_ levels were directly and positively associated with 25(OH)D_3_ concentrations, supporting the biological continuity of vitamin D metabolism in RTRs despite ongoing immunosuppressive therapy. This relationship indicates that maintaining adequate 25(OH)D_3_ status can effectively promote the synthesis of active vitamin D metabolites, mirroring mechanisms described in the general population [48]. Importantly, given the lack of association between anthropometric parameters and eGFR in our cohort, as well as the comparable prevalence of vitamin D3 deficiency to that observed in the general population [7,49], it may be reasonable to consider applying general population recommendations for vitamin D3 [50] supplementation and deficiency thresholds to KTRs in the late period after KTx with satisfying renal graft functions. Therefore, that approach could provide a practical and evidence-aligned framework for clinical management, particularly in the absence of transplant-specific guidelines supported by robust outcome data.

## 4. Materials and Methods

### 4.1. Study Design and Population

This cross-sectional study included stable KTRs who underwent kidney transplantation between 1994 and 2018 and were followed at the outpatient clinic of the Department of Nephrology, Charité—Universitätsmedizin Berlin, Germany, between February and July 2018. All participants provided written informed consent. This study was conducted in accordance with the Declaration of Helsinki and approved by the local ethics committee (EA 1/252/17). We analyzed demographic data; immunosuppression status; and available clinical information, including transplant characteristics, hypertension, diabetes, and CVD, as previously described [51]. To capture a pragmatic, real-world clinical cohort, patients were included regardless of their ongoing maintenance therapies, including various vitamin D supplementation regimens.

Demographic data, transplant-related variables, comorbidities (hypertension, diabetes mellitus, and cardiovascular disease), immunosuppressive therapy, and laboratory parameters were obtained from medical records (T-Base). The primary objective of the analyses was to assess the relationship between vitamin D status, kidney function, mineral metabolism, and body composition.

### 4.2. Laboratory Measurements

Laboratory assessments included serum creatinine, eGFR (CKD-EPI), 25-hydroxyvitamin D [25(OH)D_3_], 1,25-dihydroxyvitamin D [1,25(OH)_2_D_3_], calcium, phosphate, and parathyroid hormone (PTH). Elevated serum creatinine was defined as >1.2 mg/dL (>106.1 µmol/L), and proper kidney function was considered as eGFR ≥ 60 mL/min/1.73 m^2^. Abnormal values for other laboratory parameters were defined as 1,25(OH)_2_D_3_ < 18 pg/mL (<43 pmol/L), PTH > 65 pg/mL (>6.9 pmol/L), calcium < 8.5 or >10.0 mg/dL (<2.0 or >2.5 mmol/L), and phosphate < 2.5 or >4.5 mg/dL (<0.81 or >1.45 mmol/L).

Vitamin D status was categorized as deficiency (<20 ng/mL), insufficiency/suboptimal (20–30 ng/mL), or optimal (>30 ng/mL), according to National Institutes of Health (NIH) criteria [52]. This categorization was based on internationally accepted clinical thresholds and was applied for several methodological and clinical reasons. These cut-off values correspond to widely used definitions proposed by the NIH and the Endocrine Society, which are commonly applied in both the general population and in studies involving KTRs, and the primary stratified analysis was intentionally designed to reflect real-world clinical practice and to allow comparison between patients with clearly distinct vitamin D status.

### 4.3. Anthropometry and Body Composition

Anthropometric measurements and body composition were assessed using bioelectrical impedance analysis (InBody 170, InBody, Eschborn, Germany). Parameters included BMI, waist circumference, waist-to-hip ratio (WHR), visceral fat area (VFA), body fat mass (BFM), soft lean mass (SLM), and phase angle (PA). BMI categories followed WHO criteria. Obesity was additionally defined by WHR and VFA thresholds. In accordance with the World Health Organization (WHO) guidelines [53], BMI values were categorized as follows: underweight (<18.5 kg/m^2^), normal (18.5–24.9 kg/m^2^), overweight (25.0–29.9 kg/m^2^), and obesity (≥30 kg/m^2^). Additionally, overweight was recognized as BMI > 25 kg/m^2^, and obesity was diagnosed as BMI ≥ 30 kg/m^2^.

### 4.4. Statistical Analysis

Statistical analyses were performed using R (version 4.2.3). Continuous variables were assessed for normality using the Shapiro–Wilk test and are presented as means with standard deviations (SD) or medians with interquartile ranges (IQR), as appropriate. For comparisons between three groups (vitamin D deficiency, suboptimal, and optimal), assumptions of residual normality and homogeneity of variances were evaluated using the Shapiro–Wilk test on one-way ANOVA residuals and Levene’s test, respectively. Normally distributed variables were compared using one-way analysis of variance (ANOVA), whereas non-normally distributed variables were analyzed using the Kruskal–Wallis test. Categorical variables are presented as counts and percentages and were compared using Fisher’s exact test, with the Freeman–Halton extension applied for multi-level comparisons. Associations between clinical variables were assessed using Pearson’s or Spearman’s correlation coefficients, depending on data distribution. To identify independent determinants of graft function and vitamin D status, multivariable quantile regression models were applied due to non-normal distribution of outcome variables and heteroscedasticity, evaluating the cohort under real-world clinical conditions. All statistical tests were two-tailed, and results were considered statistically significant at *p* < 0.05. Given the exploratory nature of this study, which aimed to comprehensively screen multiple novel body composition metrics, no formal adjustments for multiple comparisons (such as the Bonferroni correction) were applied to control for Type I errors. Consequently, *p*-values associated with secondary clinical outcomes and exploratory correlations should be interpreted as descriptive and hypothesis-generating rather than definitive, minimizing the risk of Type II errors that could obscure potentially meaningful biological trends.

## 5. Conclusions

Finally, we did not find a direct relationship between anthropometric parameters, vitamin D3 status, and eGFR, indicating that changes in body composition, vitamin D deficiency, and graft function may evolve independently. This suggests that in KTRs, 25(OH)D_3_ deficiency is primarily driven by environmental and therapeutic factors rather than graft function or body composition, whereas active 1,25(OH)_2_D_3_ synthesis remains closely tied to renal function. The observed positive correlation between 25(OH)D_3_ and 1,25(OH)_2_D_3_ further suggests that cholecalciferol supplementation enhances the concentration of the active metabolite, supporting the rationale for using classical vitamin D3 in addition to active forms as in the general population. However, further studies are needed to clarify these relationships and to establish reliable, evidence-based recommendations for vitamin D management in RTRs.

### Limitations

The present study has several limitations that should be acknowledged when interpreting the results. Firstly, the cross-sectional design of the study precludes the establishment of causal relationships between vitamin D status, body composition, and renal graft function. The observed associations represent a single time-point assessment and do not allow evaluation of longitudinal changes or the direction of potential effects. Importantly, the relationship between graft function and vitamin D metabolites primarily reflects the fact that better graft function preserves renal 1-alpha-hydroxylase activity, rather than vice versa. Consequently, the results should be interpreted as exploratory rather than confirmatory. Secondly, a substantial proportion of the study population was receiving vitamin D supplementation, either in the form of nutritional vitamin D (cholecalciferol) or active vitamin D analogues. Although this therapeutic heterogeneity may introduce a confounding bias into our statistical models (such as ANOVA and regression analyses), this therapeutic intervention may have influenced circulating concentrations of vitamin D metabolites and potentially attenuated natural associations between vitamin D status and graft function. However, the primary objective of the study was not to assess supplementation efficacy but rather to analyze the relationship between vitamin D status and clinical and anthropometric parameters under real-life conditions. Excluding these patients would result in a highly selected, non-representative cohort, undermining the real-world clinical value of this study. While the inclusion of bone mineral density measurements would provide a more comprehensive assessment of the bone mineral axis, the primary objective of this study was to analyze anthropometric parameters, vitamin D3 levels, and graft function. Focusing on bioelectrical impedance analysis allowed for a detailed evaluation of the patients’ metabolic and nutritional status, which was the main research priority.

## Figures and Tables

**Table 1:** Characteristics of the study population.

	Mean (SD) Deficiency *n* = 100	Mean (SD) Suboptimal *n* = 103	Mean (SD) Optimal *n* = 112	*p*
male, *n* (%)	60 (60%)	69 (67%)	63 (56.3%)	0.32
age (y), mean (SD)	52.1 (±13.5)	52.2 (±14.2)	52.5	0.51
diabetes mellitus, *n* (%) (any type 1, 2, PTDM)	19 (19%)	21 (20.4%)	23 (20.5%)	0.41
cardiovascular disease, (CAD, PAD) *n* (%)	13 (13%)	21 (20.4%)	29 (25.9%)	0.06
hypertension, *n* (%)	91 (91%)	92 (89.3%)	90 (80.4%)	0.07
heart failure, *n* (%)	21 (21%)	26 (25.2%)	31 (27.7%)	0.51
malignancy, *n* (%)	12 (12%)	13 (12.6%)	15 (13.4%)	0.95
pre-emptive KTx, *n* (%)	10 (10.1%)	16 (15.5%)	16 (14.3%)	0.47
time after KTx, months, mean (SD)	90 (±75.1)	96.4 (±75.1)	89.6 (±74.8)	0.81
Hgb (g/dL) mean (SD)	12.7 (2.00)	12.9 (1.81)	12.8 (1.43)	0.72
CRP (mg/L) mean (SD)	5.6 (13.3)	4.3 (5.7)	4.1 (7.1)	0.48
albumin g/L mean (SD)	43.4 (3.2)	43.3 (2.9)	43.9 (3.3)	0.4
cholecalciferol supply	19 (19.00%)	36 (35%)	58 (51.8%)	<0.0001

SD (standard deviation), PTDM (post-transplant diabetes mellitus), KTx (kidney transplantation), CAD (coronary artery disease), PAD (Peripheral Artery Disease), Hgb (hemoglobin), CRP (C-reactive protein).

**Table 2:** Laboratory and anthropometric variables in patients with different levels of vitamin D3.

Variable	Deficiency *n* = 100	Suboptimal *n* = 103	Optimal *n* = 112	*p*
eGFR CKD-EPI	53.5 (38.0–70.25)	47.0 (37.5–65.0)	48.0 (34.0–64.0)	0.1641
1,25(OH)_2_D_3_ [pg/mL]	34.1 (24.0–44.0)	37.9 (27.8–52.0)	39.4 (27.5–51.8)	0.064
25(OH)_2_D_3_ [ng/mL]	15.3 (11.7–17.5)	25.0 (22.5–27.5)	36.6 (33.6–42.2)	<0.0001
PTH [ng/L]	85.25 (53.1–122)	67.7 (43.25–112.25)	49.7 (33.0–69.9)	<0.0001
Calcium [mg/dL]	9.6 (9.3–10.1)	9.6 (9.3–9.9)	9.5 (9.2–9.8)	0.75
Phosphate [mg/dL]	2.5 (2.1–3.1)	2.7 (2.1–3.1)	2.7 (2.3–3.2)	0.4
BMI [kg/m^2^]	24.8 (22.15–28.2)	25.0 (22.95–27.8)	25.1 (21.9–28.4)	0.8432
WHR	0.91 (0.85–0.99)	0.92 (0.865–0.975)	0.92 (0.84–0.98)	0.9969
WC [cm]	87.9 (80.5–101.9)	89.7 (83.0–98.65)	88.0 (80.4–100.5)	0.8249
VFA [cm^2^]	92.4 (66.5–133.9)	99.3 (70.9–135.7)	85.5 (64.2–128.3)	0.5525
BFM [kg]	19.3 (13.4–28.2)	20.9 (15.25–26.5)	18.0 (13.0–25.4)	0.4827
PA	4.9 (4.2–5.5)	4.9 (4.25–5.55)	5.0 (4.4–5.6)	0.5870
SLM [kg]	50.2 (41.8–57.3)	53.5 (43.55–59.7)	51.8 (42.4–60.9)	0.4238
Obesity	16 (16%)	12 (11.7%)	19 (17.0%)	0.53
Overweight	31 (31%)	43 (41.8%)	38 (33.9%)	0.23

eGFR CKD-EPI (Estimated Glomerular Filtration Rate—Chronic Kidney Disease–Epidemiology Project), PTH (parathyroid hormone), BFM (body fat mass), BMI (Body Mass Index), SLM (soft lean mass), WHR (waist-to-hip ratio), PA (phase angle), WC (waist circumference), VFA (visceral fat area).

**Table 3:** Immunosuppressive therapy among the study population.

Drug	Deficiency *n* = 100	Suboptimal *n* = 103	Optimal *n* = 112	*p*
tacrolimus, *n* (%)	67 (67%)	69 (67%)	64 (57.1%)	0.71
cyclosporine, *n* (%)	23 (23%)	25 (22.5%)	19 (18.5%)	0.68
betalcept, *n* (%)	6 (6%)	13 (12.6%)	14 (12.5%)	0.24
mTOR inhibitor, *n* (%)	2 (2%)	5 (4.9%)	3 (2.7%)	0.49
MMF, *n* (%)	54 (54%)	39 (37.9%)	43 (38.4%)	0.021
MPS, *n* (%)	40 (40%)	66 (64.1%)	54 (48.2%)	0.018
GCS, *n* (%)	50 (50%)	53 (51.5%)	56 (50%)	0.62

mTOR inhibitor (mammalian target of rapamycin inhibitor), MMF (mycophenolate mofetil), MPS (mycophenolate sodium), GCS (glucocorticosteroids).

**Table 4:** Results of multivariable quantile regression analysis of eGFR across different functional levels (0.25, 0.50, and 0.75 quantiles) for key clinical and anthropometric variables.

Variable	Direction of Association	0.25 Quantile (Lower eGFR)	0.50 Quantile (Median eGFR)	0.75 Quantile (Upper eGFR)
Age	Negative (↓)	*p* = 0.0003	*p* < 0.0001	*p* = 0.0018
Calcium	-	*p* = 0.68	*p* = 0.68	*p* = 0.96
Phosphorus	Negative (↓)	*p* < 0.0001	*p* = 0.0002	*p* = 0.0002
PTH	Negative (↓)	*p* = 0.006	*p* = 0.0002	*p* = 0.0011
25(OH)D_3_	Negative (↓) *	*p* = 0.039	*p* = 0.14	*p* = 0.27
1,25(OH)_2_D_3_	Positive (↑)	*p* < 0.0001	*p* = 0.0002	*p* = 0.0591
years post KTx	-	*p* = 0.36	*p* = 0.89	*p* = 0.78
BMI, WHR, WC, VFA, BFM	-	*p* > 0.05 (Not Significant)	*p* > 0.05 (Not Significant)	*p* > 0.05 (Not Significant)
SLM	Negative (↓) **	*p*≈0.39	*p* ≈ 0.16	*p* = 0.0065

PTH (parathyroid hormone), SLM (soft lean mass), BMI (Body Mass Index), WHR (waist-to-hip ratio), VFA (visceral fat area), BFM (body fat mass), WC (waist circumference). * Only in lower quantile, ** only in upper quantile.

**Table 5:** Results of multivariable regression analysis of 25(OH)D_3_ level for key clinical and anthropometric variables.

Variable	Estimate	95% CI	*p*-Value
Years post-KTx	−0.35	−0.91–0.21	0.220
Age (y)	0.048	−0.21–0.307	0.714
eGFR CKD-EPI	−0.165	−0.357–0.026	0.091
PTH	−0.039	−0.081–0.003	0.067
Calcium	−30.553	−52.719–−8.388	0.007
Phosphorus	1.882	−13.882–17.645	0.815
Tacrolimus (yes = 1)	−3.798	−13.766–6.170	0.455
Cyclosporine (yes = 1)	−1.481	−12.188–9.226	0.786
BMI	0.043	−0.674–0.760	0.906
WHR	−10.508	−44.814–23.799	0.548
WC	−0.140	−0.368–0.088	0.230
VFA	−0.016	−0.081–0.049	0.628
BFM	−0.078	−0.404–0.249	0.641
SLM	−0.030	−0.348–0.288	0.853
PA	1.590	−3.343–6.522	0.528

PTH (parathyroid hormone), BMI (Body Mass Index), WHR (waist-to-hip ratio), PA (phase angle), VFA (visceral fat area), BFM (body fat mass), SLM (soft lean mass), WC (waist circumference).

## Data Availability

Data and materials are available after contact with the corresponding author. The data are not publicly available due to privacy.

## Abbreviations

The following abbreviations are used in this manuscript:

BFM	Body Fat Mass
BIA	Bioelectrical Impedance Analysis
BMI	Body Mass Index
CI	Confidence Interval
CKD	Chronic Kidney Disease
CKD-EPI	Chronic Kidney Disease–Epidemiology Project
CKD-MBD	Chronic Kidney Disease–Mineral Bone Disorders
CRP	C-reactive protein
eGFR	Estimated Glomerular Filtration Rate
GCS	Glucocorticosteroids
Hgb	Hemoglobin
IQR	Interquartile Range
KTR/RTR	Kidney/Renal Transplant Recipients
KTx	Kidney Transplantation
MMF	Mycophenolate Mofetil
MPS	Mycophenolate Sodium
mTOR	Mammalian Target of Rapamycin
NIH	National Institutes of Health
PA	Phase Angle
PAD	Peripheral Artery Disease
PTH	Parathyroid Hormone
PTDM	Post-Transplant Diabetes Mellitus
RAAS	Renin–Angiotensin–Aldosterone System
RRT	Renal Replacement Therapy
SD	Standard Deviation
SLM	Soft Lean Mass
VDR	Vitamin D Receptor
VFA	Visceral Fat Area
WC	Waist Circumference
WHR	Waist-to-Hip Ratio
WHO	World Health Organization
